# A Novel CNN-LSTM Hybrid Model for Prediction of Electro-Mechanical Impedance Signal Based Bond Strength Monitoring

**DOI:** 10.3390/s22249920

**Published:** 2022-12-16

**Authors:** Lukesh Parida, Sumedha Moharana, Victor M. Ferreira, Sourav Kumar Giri, Guilherme Ascensão

**Affiliations:** 1 Department of Civil Engineering, Shiv Nadar University, Greater Noida 201314, India; 2 RISCO, Department of Civil Engineering, University of Aveiro, 3810-193 Aveiro, Portugal; 3 Department of CSE, Srinix College of Engineering, Gopalgoan 756003, India

**Keywords:** bond strength, concrete, deep learning, EMI techniques, machine learning, piezo sensor, pull-out, statistical indices

## Abstract

The recent application of deep learning for structural health monitoring systems for damage detection has potential for improvised structure performance and maintenance for long term durability, and reliable strength. Advancements in electro-mechanical impedance (EMI) techniques have sparked attention among researchers to develop novel monitoring techniques for structural monitoring and evaluation. This study aims to determine the performance of EMI techniques using a piezo sensor to monitor the development of bond strength in reinforced concrete through a pull-out test. The concrete cylindrical samples with embedded steel bars were prepared, cured for 28 days, and a pull-out test was performed to measure the interfacial bond between them. The piezo coupled signatures were obtained for the PZT patch bonded to the steel bar. The damage qualification is performed through the statistical indices, i.e., *root-mean-square deviation (RMSD) and correlation coefficient deviation metric (CCDM),* were obtained for different displacements recorded for axial pull. Furthermore, this study utilizes a novel Convolutional Neural Network-Long Short-Term Memory (CNN-LSTM)-based hybrid model, an effective regression model to predict the EMI signatures. These results emphasize the efficiency and potential application of the deep learning-based hybrid model in predicting EMI-based structural signatures. The findings of this study have several implications for structural health diagnosis using a deep learning-based model for monitoring and conservation of building heritage.

## 1. Introduction

Reinforced cement concrete is a composite material, obtained through naturally available sources, yields the better compressive strength and most cost-effective material, eliminates its brittle behavior with the addition of ductile reinforcing bars. To achieve composite behavior, loads must be transferred from concrete to steel through strain compatibility, attained through proper interface bonding, a load transfer that takes place near the steel–concrete interface and is idealized as a continuous stress field [1]. The performance of steel in reinforced concrete constructions depends on the interfacial frictional resistance. Early studies for bond strength measurement mostly focused around on destructive techniques using pull-out tests experimentally to assess the interfacial adhesion between reinforcement and concrete [2,3,4]. In the real world, it is essential to determine the ultimate load which can be endured by reinforced concrete before construction work takes place, i.e., removing formwork and optimum curing days [5,6,7]. Developing a method that can reliably and nondestructively evaluate the bond strength would be highly advantageous. 

In recent decades, structural health monitoring has received great attention among researchers because of its performance, reliability, and consistency in infrastructure systems [8,9,10]. Monitoring progressive decay is an essential component of SHM systems. SHM techniques may significantly lower maintenance costs and improve structural stability. The use of piezoelectric materials with aid to SHM has recently increased in reliability testing and preventive maintenance of the structure [11]. Yu et al. [12] proposed a temperature sensor to measure the resonance wavelength. The reliability of the sensor works with good accuracy and is convenient to use. Ye et al. [13] designed a novel PEC sensor to detect the trace quantity of Cu^2+^. The potential of piezoelectric materials for sensing and actuation purposes is a widely recognized as distinctive feature, required for better SHM [14]. An emerging approach to evaluate the performance of SHM techniques has been shown in a wide spectrum of constructions [15,16,17,18]. Liang et al. [19] first developed one dimensional (1D) EMI-based measurements and structural impedance-based parameters, which is useful for structural identification. 

Many past studies have focused on the applications of EMI techniques, among others, in concrete structures [20,21,22], bond slip [23], corrosion [24], debonding damage [25], wind turbines [26], fatigue [27], and damage identifications [28,29]. Structural health assessment of concrete structures to track the evolution of strength properties has also proven the viability through EMI approach. Gu et al. [30] have made a significant contribution using a periodic response frequency for local-based SHM technique, i.e., EMI technique. The cement hydration effect was monitored using EMI techniques as an indication of bond development among conventional concrete and rebars [31]. Further, EMI techniques were also explored to understand the bond-slip between concrete-composite structures [32]. Jiang et al. [33] draws on an extensive range of sources to use active-sensing-based sensor for bond loss monitoring between fiber reinforced bars and concrete. Key innovations in this area non-destructive testing (NDT)-based EMI techniques to measure the bond strength between steel and concrete for load deformation levels. In real-time situations and design implication, for serviceability approach the bond stress between the concrete and reinforcement only measures under elastic load not to ultimate approach. 

Artificial neural network (ANN) is a potent tool, integrated with various SHM techniques to assess the damage location and quantification for long term strength and durability check for infrastructures. The deep learning approach with adaptive learning ability made significant advancement among researchers for concrete strength and damage prediction. The ANN framework has been used to forecast the physical characteristics of concrete strength, corrosion, tensile strength, and bond development, etc., and the accuracy of this method ensured with a mean absolute percentage error (MAPE), RMSE, and RMSD [34]. Supervised learning has been used by researchers as a better algorithmic approach for classification problems, including identifying deterioration in civil constructions [35]. In recent past, several studies have implemented machine learning (ML) techniques successfully applied to various SHM-based structural applications such as structural damage detection [36], arch bridges [37], composite structures [38], wind turbines [39], corrosion [40], and steel frames [41]. In the field of civil engineering, mostly ML approaches are still scarce and only limited to image classification and digital image correlation [42]. Several SHM implementations have been reported that focus on multilevel perceptron and recurrent neural network methods [43,44]. Eventually, new classes of NN emerged, including fuzzy ARTMAP networks (FAN) and probabilistic neural networks (PNN), due to their consistent performance, better accuracy, and lower processing time [45,46,47,48]. In last decade, several studies used to identify structural deterioration in SHM systems based on PNN and FAN have been previously discussed [49,50,51]. Before extensive utilization of deep learning in SHM systems, classification models, including the support vector machines (SVMs) [52], principal component analysis (PCA) [53], and k-means [54] methods, were used to extract data characteristics. 

In a recent development, the convolutional neural network (CNN), which has received plenty of research and has been used in practical applications, is one of the most significant end-uses of deep learning in SHM approaches. CNN is considered one of the most efficient and successful feature selection methods. It has been employed in numerous fields such as remote sensing [55], image recognition [56], damage detection [57], vibration-based structural state [58], civil infrastructure [59], bolt looseness [60], disease [61], and rock classifications [62]. Several studies have suggested using image processing methods with convolutional neural networks to identify cracks and corrosion for civil and mechanical infrastructure [63,64,65]. These methods can accurately recognize, locate, and visualize cracks and corrosion [66,67]. One-dimensional CNN is considered to be a fast and accurate solution for the early detection of structural joints [68]. CNN is a potential tool to identify extensive damage using impedance-based SHM in simple and direct damage scenarios [69]. However, few studies have explored deep CNN using EMI signals for damage diagnosis assessment in interfacial damage and debonding. Recently, a single CNN framework with a video processing focus appeared in the SHM domain [70]. In [71], the researchers proposed the monitoring and setting time of cement hydration using the EMI-IntNet model. Similarly, in [72], a deep learning-based model was proposed, which significantly reduces the complexity of EMI inversion and enables quick and precise subsurface early mild cognitive impairment (EMCI) estimate from multi configuration EMI signals.

In last few decades, the research primarily focused on the prediction of bond strength using regression analysis. Concha [73] considered neural network model to predict the bond between fiber reinforced polymer and concrete. Alizadeh et al. [74] presents a neuro fuzzy technique to predict the behavior of composite bar and concrete. Several other studies utilized a machine learning approach to predict the bond strength between reinforcement and concrete [75], corroded reinforcement concrete [76], FRP-concrete [77], ultra-high performance concrete-bar [78], and fiber-reinforced cement mortar concrete [79]. Moreover, in the recent past, other soft computing methods were used to predict spliced GFRP bars and concrete beams [80].

From the above discussion, it is found that the implication of the CNN-based deep learning algorithm for frequency-based monitoring, e.g., impedance spectroscopy over the higher frequency is very high. None of the past studies include hybrid-based features extraction algorithm for piezo impedance-based sensor signals. This motivates the authors to implement the deep learning-based hybrid models to monitor the steel–concrete interaction using piezo electric sensor for EMI technique. This paper aims to find the feasibility of the PZT patch for monitoring the concrete-steel interfacial bond through a piezo coupled admittance signature. The damage indices, i.e., RMSD and CCDM indices, were plotted for damage quantification. This study also proposed a novel hybrid (CNN-LSTM) model for a baseline prediction that combines the EMI-PZT-based signals with the deep learning algorithm. Further, it is extended to forecasting the signatures (i.e., structural peaks and piezo-resonance peaks for conductance signature) for more accurate prediction of concrete-steel bond degradation. Overall, this paper focuses more on the sensitivity and employability of a proposed hybrid model to predict and forecast the baseline structural signature for the statistical damage identification and bond strength monitoring of a piezo coupled signature.

## 2. Materials and Methods

This section included materials, experimental design, and deep learning models for the prediction of baseline signature and bond strength forecasting for lab sized reinforced concrete samples. The hybrid model used in this study is a combination of long short-term memory (LSTM) [81] and CNN [82], found to be suitable for the prediction of sequence data. A novel hybrid model known as CNN-LSTM is proposed for this intended study, whose performance in terms of metrics such as mean absolute percentage error (MAPE), mean absolute error (MAE), and root mean squared error (RMSE) are analyzed, compared, and discussed for insightful information towards bond strength. To assess the reliability of the EMI-CNN-LSTM model, the investigated bond strength vs. slip using pull-out tests were also performed in static manner. The PZT instrumented with a steel bar facilitates the indication bond strength and it is measured through piezo-coupled signatures. The detailed fabrication of the sample, methods, and materials is presented as follows.

### 2.1. Fabrication of Samples

**Figure 1:**
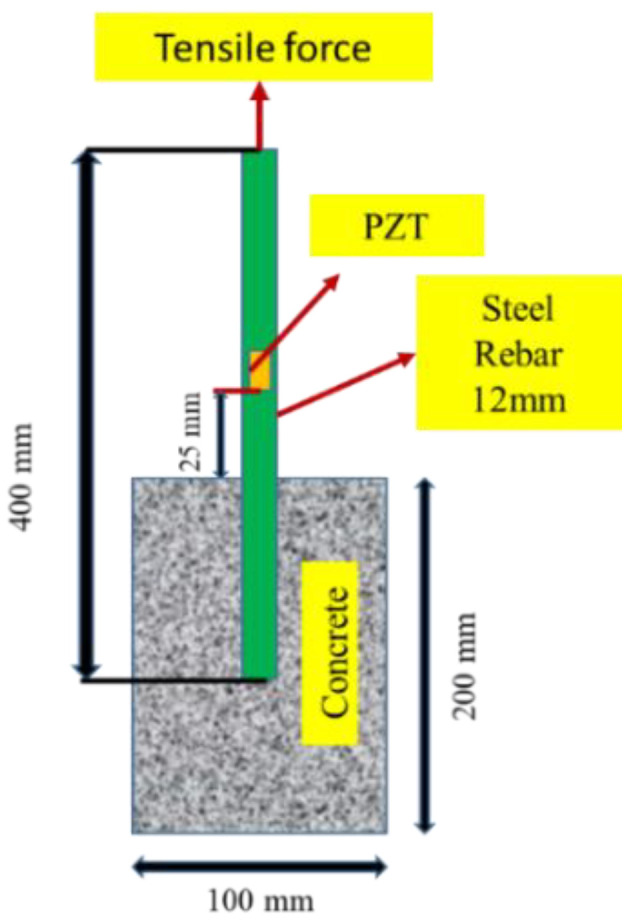
Specimen dimensions with PZT location.

Three cylindrical samples were prepared to investigate the bond strength among concrete and reinforcing bar. The dimensions of the specimen were 100 mm×200 mm. Figure 1 shows the detailed dimensions of the specimen. Three different cylindrical samples are tested, and the observations are indicated as the mean of the three samples (see Section 4.1.

### 2.2. Materials

The raw materials used for the casting of lab specimens are OPC 43 cement, river sand, coarse aggregate, and water. The specific gravity and water absorption of coarse aggregates was measured to be 3.15 and 1.67%, respectively. The size gradation of aggregates was performed by IS383:1983 [83]. 

**Figure 2:**
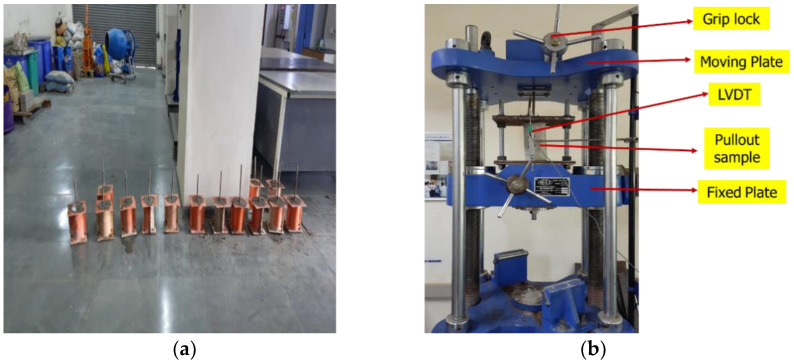
(**a**) Casting of cylinder specimen, (**b**) sample mount in the universal testing machine UTM.

The steel bars were of 12 mm dia with yield strength of 500 MPa conforming to IS 1786 (2008) [84]. Cement concrete mix design was adapted from BIS 10262:2009 [85], and were embedded in the concrete for lab-cast cylindrical samples (see Figure 1). The adopted water-to-cement ratio was 0.55 based on mix design codal values [85]. Table 1 provides the mix proportioning of concrete specimens. The concrete mixing and placing procedures for the lab size are illustrated in Figure 2. In this study, the pullout test experiments were conducted for reinforced concrete samples after completion of 28 days curing. 

### 2.3. Pull-Out Specimen Details

The pull-out samples were prepared according to ASTM C234 [86], which specifies the use of 150 mm cylindrical molds for rebar sizes greater than 12 mm. The samples were prepared with concrete cylinders with a single reinforcing steel placed in the center of the sample. As seen in Figure 1, the bar was extended upward by nearly 350 mm from the top of the cylinder to provide enough length to grip to jack of the testing apparatus. The embedded length of steel was kept 50 mm into the concrete for bond strength development. The molds were cast in three layers and were vibrated on the vibrating table for 15–20 s until proper compaction was attained. The top level was leveled to create a smooth finish. After casting, the samples were wrapped in plastic enclosed to prevent loss of moisture. After 24 h, the samples were demolded and kept for pond curing for 28 days at room temperature and humidity.

### 2.4. Pull-Out Testing

The standard pull-out test was performed for all three concrete samples. The loading rate was kept 0.1 kN/s. The integrated UTM data acquisition system was attached to a high-accuracy linear variable of differential transducers (LVDTs) to monitor the slip of the rebar against the load/stress applied. The samples were encased between two plates, and leather sheets were placed between the plate and the surface of the samples to guarantee an evenly distribution of loading pressure across the samples’ surfaces. The total deformation for slippage and steel expansion was acquired. The load was applied until it reached the ultimate peak load, and the rebar detached from the sample. The maximum bond strength was determined using Equation (1).

(1) $$\tau =\frac{P_{u}}{\pi l_{d}d_{b}}$$

where τ denotes the maximum bond strength, $P_{u}$ ultimate peak load of the steel rebar, $d_{b}$ diameter of steel bar, and $l_{d}$ embeddedment length of rebar.

### 2.5. EMI Techniques Setup

EMI observations were recorded along with the pull-out test via LCR meter (Agilent 3441E). The square PZT patch of dimension (10 × 10 × 0.3 mm^3^) was bonded to the steel bar in each of the samples at a distance of 20 mm from the upper part of the concrete (see Figure 1). The selected spacing ensured for the adequate sensing radius of the PZT patch varied from 0.4 to 0.45 m for reinforced concrete structures [87]. The properties of PZT patches are given in Table 2. The PZT patches were assembled to the steel surfaces with an Araldite adhesive. The physical properties of the steel and adhesive are listed in Table 2. 

**Figure 3:**
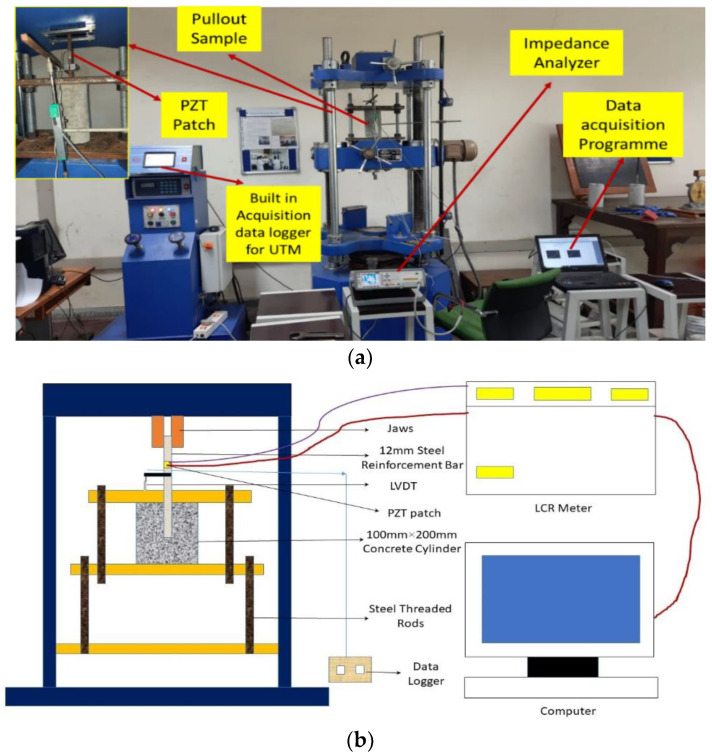
(**a**) The experimental setup for bond strength monitoring (**b**) Schematic diagram.

The flat PZT patch was connected to the machined steel surface through a uniform coating of adhesive paste over it. Gentle pressure was applied to the upper surface of the PZT patch with the palm for few minutes, and then it was left untouched for at minimum 24 h at ambient temperature to allow the adhesive to cure completely. To perform the EMI readings, the electrical wires were soldered to the top layer of the PZT patch and joined to the impedance analyzer. The admittance signatures were monitored using an impedance analyzer with a frequency range between 30 kHz and 300 kHz. Figure 3a,b illustrates the experimental setup for cylindrical samples performed in this research. LVDT measures the slip of the bar from the concrete during the experiment. The LVDT-attached loaded end of the bar included the slip due to deformation of the bar and concrete. Hence, it is necessary to consider these aspects for calculating the slip of the bar.

(2) $$\mathrm{Slip}=LVDT\;reading-\delta_{c}-\delta_{s}$$

(3) $$\delta_{c}=\frac{P\cdot l_{c}}{A_{c}\cdot E_{c}}$$

(4) $$\delta_{s}=\frac{P\cdot l_{f}}{A_{s}\cdot E_{s}}$$

where *P* = applied load, $A_{s}=$ Area of steel, $E_{s}\;$= Elastic modulus of steel, $A_{c}\;$= Area of concrete cylinder, $E_{c}\;$= Elastic modulus of concrete, $l_{f}\;$= free length of steel.

### 2.6. LSTM Model

**Figure 4:**
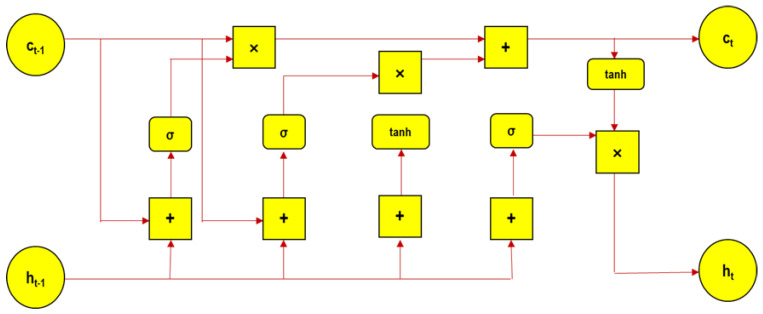
LSTM architecture.

LSTM is a sequencing and recurrent artificial neural system that consists of both long-term memory (LTM) and short-term memory (STM) processed with a series of gates. A memory cell known as a ‘cell state’ plays a central role in an LSTM model that maintains its state over a time span. Figure 4 illustrates the detailed architecture of the LSTM cell.

The current section elaborates the steps involved, to start with forgetting gate computes, the useful bits, and data flow for the current cell state considering the current input and previous hidden state. The vector is formed through new input information and the prior hidden layer in the neural network (ANN-LSTM) system, where matrix elements are normalized in the range of 0, 1. Hence, the forget gate allows us to classify the parameters/components to relevant (1) and irrelevant (0). The further process includes sharing the input data to the LSTM cell. A new memory vector is formed with the merging of previous data and current input data through the function *tanh*. The activation function sigmoid is used for the input gate, acting as a filter, and yields the relevant data for post processing. Then, the new memory vector is multiplied pointwise by the output of the input gate for the final prediction. 

Equations (5)–(10) depicts various operations involved in an LSTM cell.

(5) $$i_{t}=\sigma \left(W_{i}⊗x_{t}+U_{i}⊗h_{t-1}+b_{i}\right)$$

(6) $$f_{t}=\sigma \left(W_{f}⊗x_{t}+U_{f}⊗h_{t-1}+b_{f}\right)$$

(7) $$o_{t}=\sigma \left(W_{o}⊗x_{t}+U_{o}⊗h_{t-1}+b_{o}\right)$$

(8) $$c_{t}^{\prime }=tanh\left(W_{c}⊗x_{t}+U_{c}⊗h_{t-1}+b_{c}\right)$$

(9) $$c_{t}=f_{t}\cdot c_{t-1}+i_{t}\cdot c_{t}^{\prime }$$

(10) $$h_{t}=o_{t}\cdot \tanh\left(c_{t}\right)$$

where $i_{t}$ input gate, $f_{t}$ forgot gate, $o_{t}$ output gate, $c_{t}^{\prime }$*,* $c_{t}$ cell state, $h_{t}$ hidden state, $W_{i}$ weight for input gate, $x_{t}$ input data, *h*_*t*−1_ previous hidden state, $W_{o}$ weight for output gate, $W_{f}$ weight for forgot gate, *b* bias.

### 2.7. CNN Model

**Figure 5:**
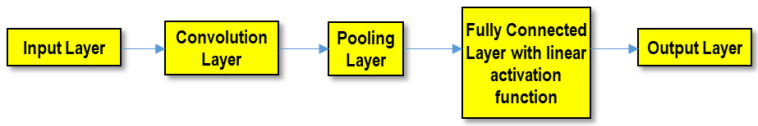
CNN Architecture.

CNN is a deep learning algorithm integrated with neural network, which primarily works on convolution theory. Due to its high accuracy, CNN model initially attracted attention for image recognition and classification. The fundamental component of a CNN model is the convolutional layer. The important parameters of this layer are a set of filters or kernels, which are responsible for the convolution operations. The output of convolution operations, which are linear in nature, are passed through non-linear activation function. The rectified linear unit (ReLu) [88] is a commonly used non-linear function in CNN as it reduces the exponential computation overhead required to operate the neural network. Followed by the convolutional layer, the pooling layer receives the output from the convolutional layer for dimensionality reduction, further eliminating the computer processing hassles. Dropout layers are incorporated at input layers or other hidden layers which nullifies the contribution of a number of neurons. A single node fully connected layer with a linear activation function receives the output from the final pooling layer. Figure 5 shows the CNN architecture for continuous output.

### 2.8. Proposed CNN-LSTM Hybrid Model

**Figure 6:**
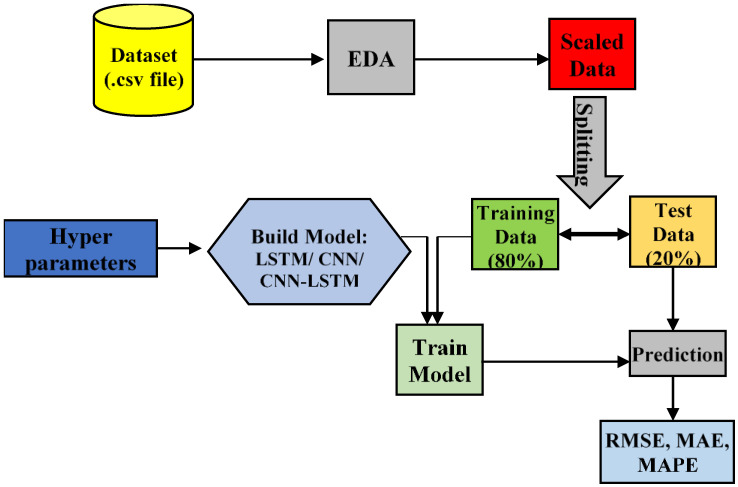
Workflow of proposed deep learning model.

The presented CNN-LSTM model combines CNN and LSTM for building the predictive model for the structural baseline signature. The first component of the hybrid model, is CNN model, needed for the feature extraction and the second component, LSTM exhibits the sequence learning. Figure 6 shows the workflow diagram of various deep learning models used in this study. As depicted in Figure 6, the data set is pre-processed through Exploratory Data Analysis (EDA) followed by data scaling. The scaled data are split into two units viz, training dataset and test dataset in the ratio 8:2. The training dataset is used for training different deep learning models, i.e., LSTM, CNN and the proposed CNN-LSTM hybrid model using optimized hyper-parameters to yield predictive models. These predictive models are used to predict the values in the test dataset range. Finally, the predicted values of the models are compared with actual test data to evaluate the model performance in terms different evaluation metrics, such as MAE, RMSE, and MAPE. The detailed descriptions are explained in Section 3.

**Figure 7:**
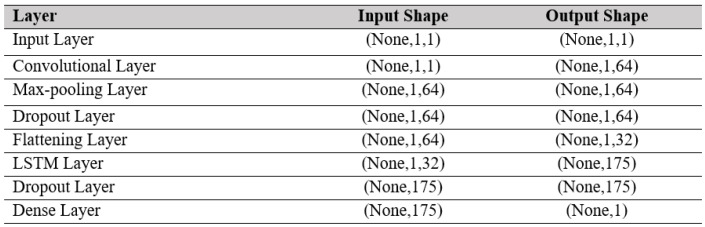
Working structure of proposed CNN-LSTM hybrid model.

Figure 7 shows the working structures of the proposed CNN-LSTM model. The proposed CNN-LSTM model consists of a CNN unit followed by an LSTM unit. In Figure 7 it shows the shape of input and output data at each processing layer in the proposed hybrid model. Two 1′s in the input data shape represent number of frequency steps and number of features, respectively. In this study, only one feature, i.e., frequency, predicts the output, i.e., conductance values of EMI signature. The convolution layer, the heart of CNN unit which has filters of size 64 and kernel size 1 in this case, receives data from the input layer. It is followed by a max pooling layer for dimensionality reduction. A dropout layer follows the max pooling layer to pass data to the next layer by dropping irrelevant information. The output of the dropout layer is then fed into the flattened layer to yield the final output of CNN unit. The LSTM layer receives input from the flattening layer which uses 175 no neurons. Another dropout layer follows to drop irrelevant information resulted from the output of the LSTM layer. Finally, a dense layer terminates the proposed CNN-LSTM architecture, which results in the final output of the model. In this proposed architecture, 1-D CNN unit is responsible for feature extraction and the LSTM unit is attributed for sequence data prediction.

## 3. Developed Method

DL methods performed in this research are implemented using Python in Jupyter notebook and executed on a Windows 11 machine with Intel(R), core-i3–8th Gen CPU and 8 GB RAM. All three models used in this work are executed separately with two samples of data lab sized concrete samples. The values obtained from the predicted result are compared with the original values present in the test data to produce different model evaluation metrics such as RMSE, MAE, and MAPE. This section includes data pre-processing, hyper parameter tuning, and performance metrics.

### 3.1. Data Pre-Processing

**Figure 8:**
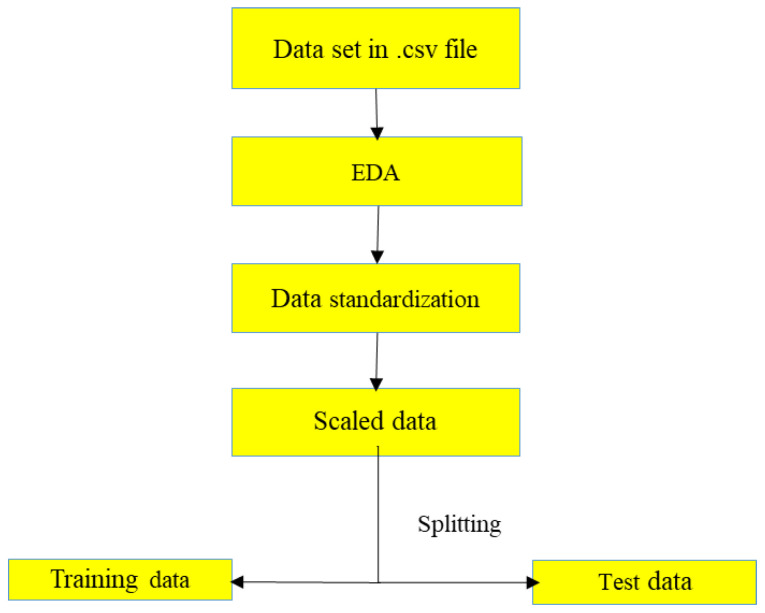
Data pre-processing.

Exploratory data analysis (EDA) was performed for the output data set to check outliers and other anomalies present in the dataset. Figure 8 depicts the workflow of data pre-processing performed on the dataset used for this study. The conductance values obtained for a given frequency range of 30 kHz to 300 kHz were obtained and processed for LSTM, CNN, and hybrid model for predicting signature to imply the bond strength development between steel and concrete.

### 3.2. Hyper Parameter Tuning

Hyper parameters [89] were used for building the LSTM and CNN models are optimized through Bayesian hyper parameter optimization technique using the ax tool [90]. The optimized hyper-parameters obtained are used to build the CNN, LSTM and CNN-LSTM hybrid model. Table 3 depicts the optimized hyper-parameters for different models used in this work.

### 3.3. Model Evaluation Metrics

Different statistical evaluation metrics used for bond strength monitoring and prediction are root mean squared error (RMSE), mean absolute error (MAE), and mean absolute percentage error (MAPE). The evaluation of metrics is useful to quantify the performance of the different DL models. Table 4 depicts different evaluation metrics to evaluate the deep learning models used in this work. 

## 4. Results and Discussions

For reinforced concrete composite construction, the strength of the interfacial bond plays a key factor for the transfer of stress between steel and concrete. Therefore, the mechanism of the interfacial bond strength is of the utmost importance to understand the behavior of crack, bearing capacity, and durability of reinforced concrete structures.

This section discusses the observations of an examination of the EMI signatures (real and imaginary parts) for bond strength measurement between rebar and concrete, which is further processed for forecasting of baseline and future EMI signatures using proposed DL models. The signatures, obtained for the experimental and predicted method, further verified statistical indices. The signatures are acquired to monitor bond strength for intermediate strain range/displacement levels for the stress and slip curve. Finally, the results of the experimental results and the predictions of the baseline EMI data using the CNN-LSTM hybrid model are analyzed and compared.

### 4.1. EMI Response Spectra

Through a mechanical impedance analyzer, the admittance signatures of the PZT patch were obtained as a function of frequency. The experiments were carried out in a repetitive manner to check sensor stabilization and for the elimination of noise and error. In past studies, the conductance signatures were successfully utilized to capture the strength and damage of the intended structure [91,92,93]. Therefore, this study only focused for obtained sensor-based conductance signature for the pull-out test for different displacement/strain ranges for a better indication of bond strength. The susceptance signature is more sensitive to temperature variations and can be utilized to check the viability of coupled PZT patches [94]. In this investigation, it was assumed that the bonding parameters and the quality of the PZT patch would not deteriorate during the measurement duration. Hence, the susceptance signatures were not taken into account for this study. 

**Figure 9:**
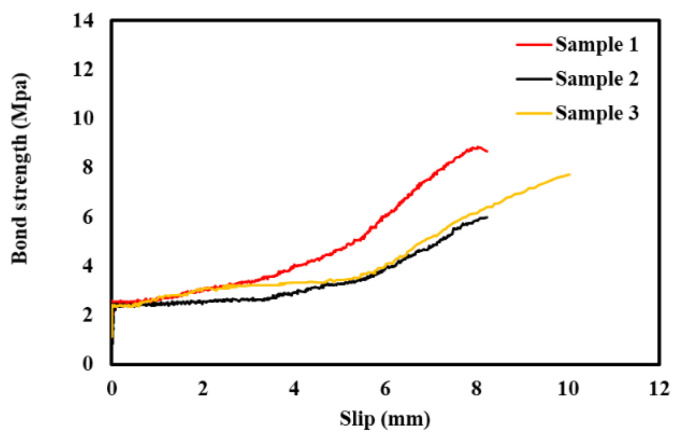
Test results for bond strength vs Slip curve.

The static pull-out test results were recorded for three different lab-sized concrete cylindrical samples embedded with steel bars. Figure 9 shows the plot between the bond strength vs slip for all the three samples. The bond strength for the three samples increases linearly up to the maximum strength until sudden failure. An 8 to 10 mm slip was observed for all samples. The fundamental bond stress distribution generally has a plateau once it reaches the ultimate load. Moreover, the interfacial bond strength between steel and concrete is lost due to adhesion and mechanical bearing. Similar trends have been reported by [95,96,97,98] in their work for normal and light-weight concrete. The piezoelectric sensor has dual sensing mechanism which allow the sensor to actuate and sense concurrently while sensor structure interaction occurs. Integrating piezoelectric sensor for EMI technique where piezoelectric charge density (hence electrical impedance) is measured. As the PZT patches (commercial version piezoelectric sensor) has very small mass inertia compared to the host structure, and the sensor signature (obtained as mechanical impedance) has constrained only host structure information. This usually reflects in the conductance (inverse of impedance) signature. For this paper, the same concept has been applied to understand the employability of a piezoelectric sensor for monitoring the bond strength of concrete and steel through a pull-out test. The yielded conductance signature obtained for different displacement control signifies the interface bond behavior (hence the strength) while experience tensile pull. The signatures can be further idealized for mass, spring, and damping system to find out how the contact stiffness (hence elastic bond behavior between concrete and steel) degrades through a progressive pulling action. Bhalla and Soh [99] derived expressions to find the admittance signatures of the surface-bonded PZT patch.

(11) $$\bar{Y}=G+Bj=4\omega j\frac{l^{2}}{h}\left[\bar{\varepsilon_{33}^{T}}-\frac{2d_{31}^{2}\bar{Y^{E}}}{\left(1-\mu \right)}+\frac{2d_{31}^{2}\bar{Y^{E}}}{\left(1-\mu \right)}\left(\frac{Z_{a,eff}}{Z_{S,eff}+Z_{a,eff}}\right)\bar{T}\right]$$

From this expression, B and G are the conductance and susceptance, l, w, h geometry of PZT, µ poisons ratio, $\bar{\varepsilon_{33}^{T}}$ complex electrical permittivity, k is wave number, $Z_{a,\mathrm{eff}}$ and $Z_{S,eff}$ denotes effective impedance PZT patch and structure, respectively. $\bar{Y^{E}}$ and $\bar{\varepsilon_{33}^{T}}$ is the complex Young’s modulus of elasticity and complex electrical permittivity, respectively. $d_{31}^{}$ Piezoelectric strain coefficient $\bar{T}$ is equal to$\frac{\tan kl}{kl}$ is called complex tangent ratio, ω is the angular frequency of excitation.

**Figure 10:**
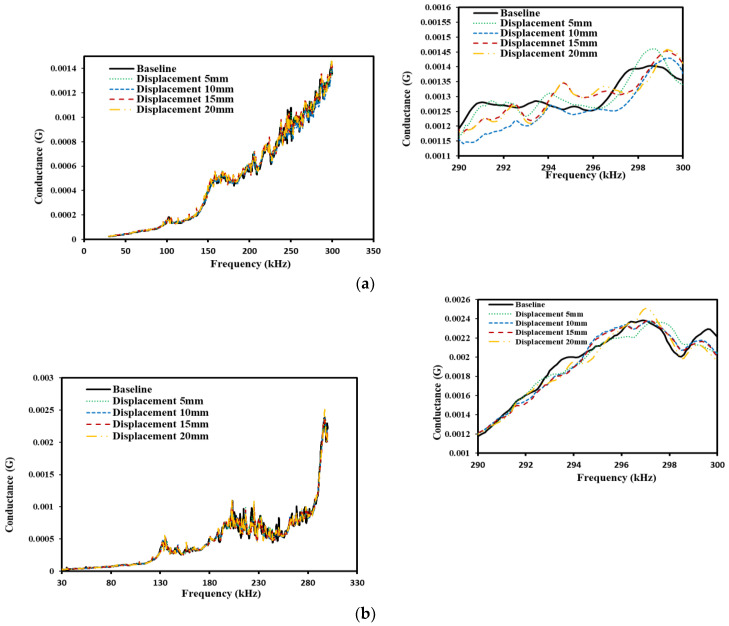
Variation of conductance signature at different displacement level, (**a**) Sample 1, (**b**) Sample 2.

Figure 10a,b represent the conductance plot acquired from the sensor-based dynamic measurements, where the PZT patch is bonded to the rebar for two lab sized concrete cylindrical samples, i.e., Samples 1,2, respectively. Figure 10a,b shows the peaks of the conductance shifts rightward with the increase in the displacement/strain ranges till the specimen fails in the splitting tensile. It is promising to observe how the variations in the conductance signatures correlate with different strain/displacement holds till they attained the maximum value. As the loss of bond over time during loading occurred, the same was reflected in EMI spectra obtained from the PZT patch. The shifting of peak frequency in EMI spectra are caused due to the failure of the interfacial transition zone between the aggregate and steel for strain/displacement range. Similar results were also reported by Soh and Bhalla [100] to monitor the curing of concrete in experimental study. From the above results, it is quite noticeable that the sensor readings are capable of monitoring bond strength between steel and concrete. Further, the static strength performs the outlay of the elastic variation of the lab-sized sample.

**Figure 11:**
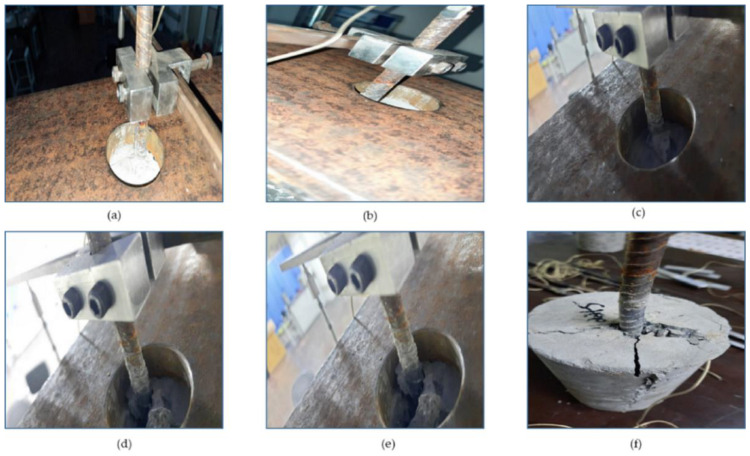
Different stages of failure at a displacement of (**a**) 0 mm, (**b**) 5 mm, (**c**) 10 mm, (**d**) 15 mm, (**e**) 20 mm, (**f**) Splitting.

**Figure 12:**
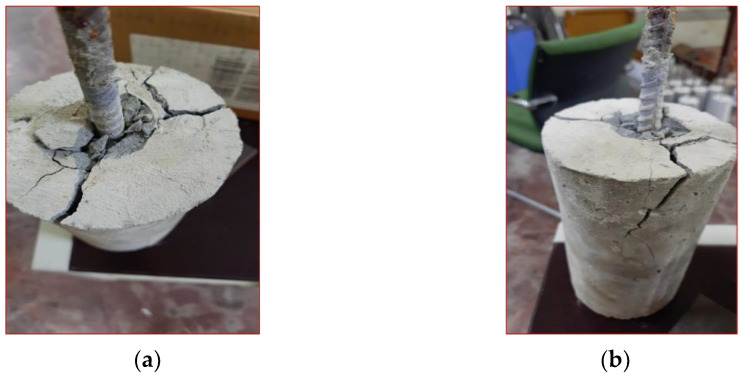
Different views of failure mode (**a**) Top, (**b**) Side.

Figure 11a–f show the typical failure pattern of bond strength between steel and concrete for different displacement/strain range outturned through axial pull-out test. The slip induced due to splitting failure was clearly visible as an indication of bond loss. Figure 12a–c shows different orientation of splitting failure in the concrete cylindrical sample. The splitting failure is more apparent due to the embedment length and deformed surface of the steel rebar. Overall, these results suggest that the failure is limited to the crack core around the steel rebar.

### 4.2. Statistical Indices 

Statistics indicators i: e RMSD [101] and CCDM [102], which are frequently used in predictive maintenance of structures as damage indices, is used to process EMI spectra to quantify the bond strength at different strain/displacement range through pull-out loading. The RMSD index for the admittance signatures is presented in Equation (11).

(12) $$\mathrm{RMSD}(\%)=100\times \sqrt{\frac{\sum_{j=1}^{N}{\left(G_{j}^{i}-G_{j}^{1}\right)}^{2}}{\sum_{j=1}^{N}{\left(G_{j}^{1}\right)}^{2}}}$$

where $G_{j}^{1}$ real part of the baseline signature for various displacement level $G_{j}^{2}$ real part of the signatures after pull-out loading at various displacement levels.

The CCDM index related to the correlation coefficient for piezo-coupled signatures is presented in Equation (12).

(13) CCDM=1−cov [Re(Gj1),Re(Gji)]σ1σ2

where *cov* is the covariance between the $G_{j}^{1}$ and $G_{j}^{2};\;\sigma_{1}{\mathrm{and}\;\sigma }_{2}$ are the respective standard deviations of each signature.

**Figure 13:**
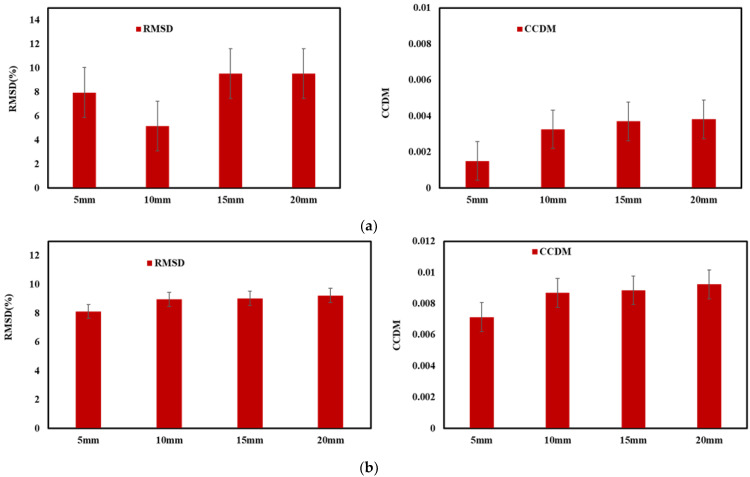
Statistical variations at different displacement levels (**a**) Sample 1, (**b**) Sample 2.

Equations (7) and (8) can be used to quantify the statistical variation of EMI signature of structural variation. Larger variations in the conductance signatures lead to higher values of the RMSD and CCDM index values when compared to baseline signature. As shown in Figure 13a,b, the RMSD and CCDM values increase with the pull-out load and are more sensitive at a higher value of displacement for Sample 1,2, respectively. This behavior indicates loss of adhesion between reinforcement and concrete and indicated the failure occurs at for different strain (and displacement) range from 0 to 20 mm. The statistical analysis shown also indicate that bond strength nondestructively assessment through EMI signals alone. A significant disadvantage of using statistical indices is the need to establish baseline measurements, which can be challenging for preexisted structure. The challenges occur for the existing structure that are vulnerable to corrosion and the baseline signatures not available to employ these techniques.

### 4.3. Deep Learning Model for Prediction of Baseline Bond Strength Data

**Figure 14:**
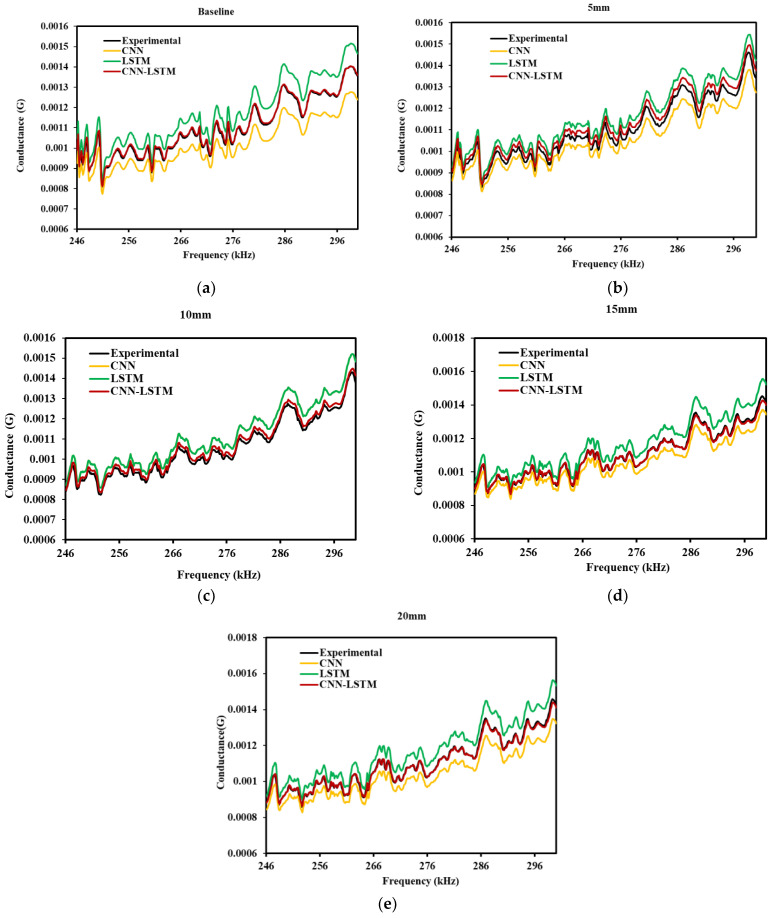
Experimental and predicted deep learning models comparison of Sample 1 (**a**) Baseline, (**b**) 5 mm, (**c**) 10 mm, (**d**) 15 mm, (**e**) 20 mm.

This section comprises a proposed deep learning model based on the experimental EMI signals described above. The EMI signals are classified for dataseta of two lab-sized samples for different strain holds during an axial pull test. To build predictive models, LSTM, CNN, and CNN-LSTM hybrid bases algorithms are developed. The optimized hyper parameters for the hybrid model are summarized in the Table 4, for precise and reliable prediction of EMI signals. The entire data set of both samples are divided separately into the training data unit and the test data unit confirming the 8:2 split ratio. The training data set is used for model fitting, and the test data set is used to validate the model. The training and validation loss confirm the proper fitting and validation. Figure 14a–e shows the variation of actual experimental conductance signature and the predicted conductance curve for the LSTM, CNN and CNN-LSTM model for sample 1. 

**Figure 15:**
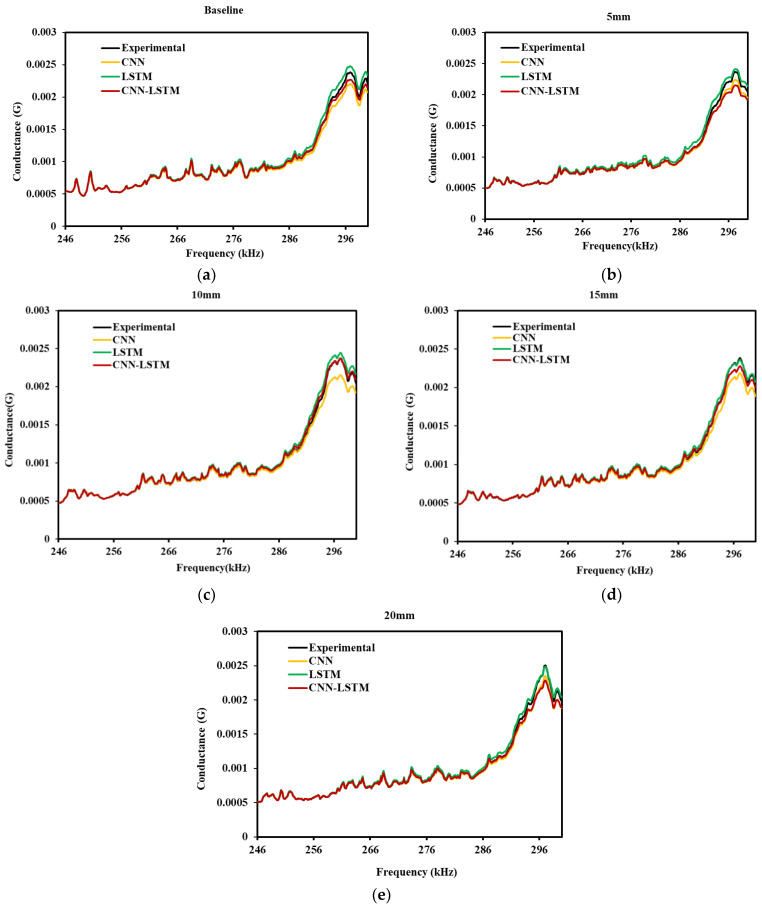
Experimental and predicted deep learning models comparison of Sample 2 (**a**) Baseline, (**b**) 5 mm, (**c**) 10 mm, (**d**) 15 mm, (**e**) 20 mm.

It is evident from Figure 14a–e that the prediction of EMI signature through different models are sufficient to identify the structural peak and piezo resonance peaks, which are crucial for impedance-based structural health monitoring. The predicted curved are completely identical with similar conductance peaks, and also follow the similar pattern for different strain range. In a closer look, it can be seen that the proposed CNN-LSTM is very close to the actual experimental result (refer Figure 14c–e). Curves are aligned, which confirms that the predicted result from the similar variation is also noticed for Sample-2 (seen in Figure 15a–e).

**Figure 16:**
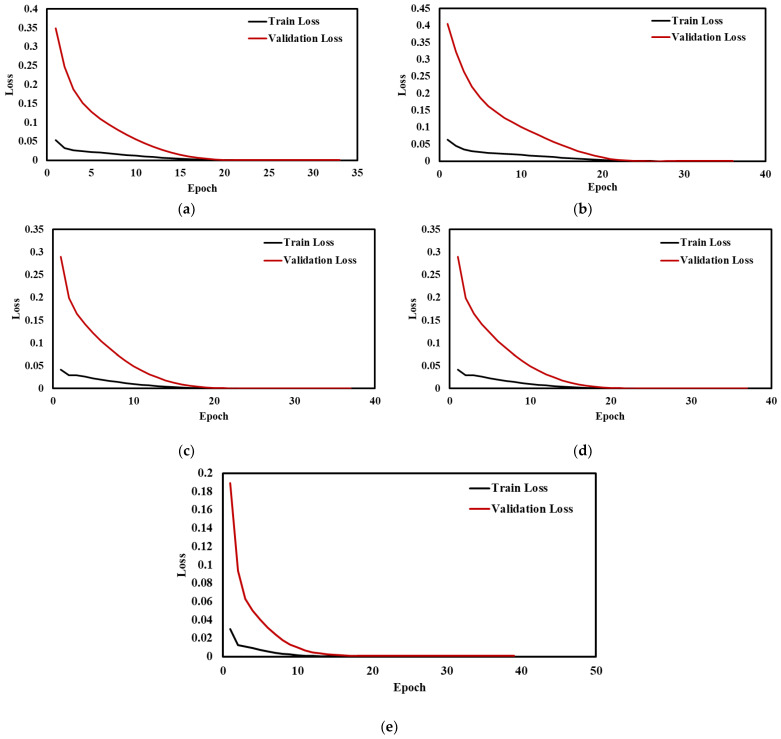
Training loss and validation loss vs No of epochs at (**a**) Baseline (**b**) 5 mm (**c**) 10 mm (**d**) 15 mm (**e**) 20 mm.

Training loss provides information about the performance of model fitting with training data, and validation loss provides information about the feasibility of model fitting with new data, further used to forecast the new results. Figure 16a–e shows the train and validation loss of the proposed CNN-LSTM hybrid model with a different number of epochs. The results show that the losses are minimized for both training and validation of datasets. Table 5 and Table 6 depict the evaluation metrics for the results obtained from LSTM and CNN model, implemented for Sample 1,2 data, respectively. Table 5 shows the performance metrics of the proposed CNN-LSTM hybrid model for Samples 1,2, for better comparison and highlights the suitability and accuracy of the proposed model. From Table 5, it can be seen that the MAPE values of the proposed CNN-LSTM model for different displacement/strain range are far superior to the MAPE values for the LSTM and CNN models, which are depicted in Table 6 and Table 7 for lab sized concrete cylindrical Sample 1,2, respectively. For Sample 1, the MAE value is 0.0000758 for the base-line case using the LSTM model and 0.0000785 using the CNN model. The MAE value of 0.0000090 for the same baseline case confirms that the result is more promising than the CNN and LSTM models. A low value of MAPE indicates minimum error and maximum accuracy for a model. Hence, the proposed CNN-LSTM model with minimum MAPE is considered to be better, compared to standalone CNN and LSTM model. Similarly, for all the other cases, it was verified that the proposed CNN-LSTM hybrid model outperforms the LSTM and CNN model with respect to the RMSE, MAPE, and MAE values.

**Figure 17:**
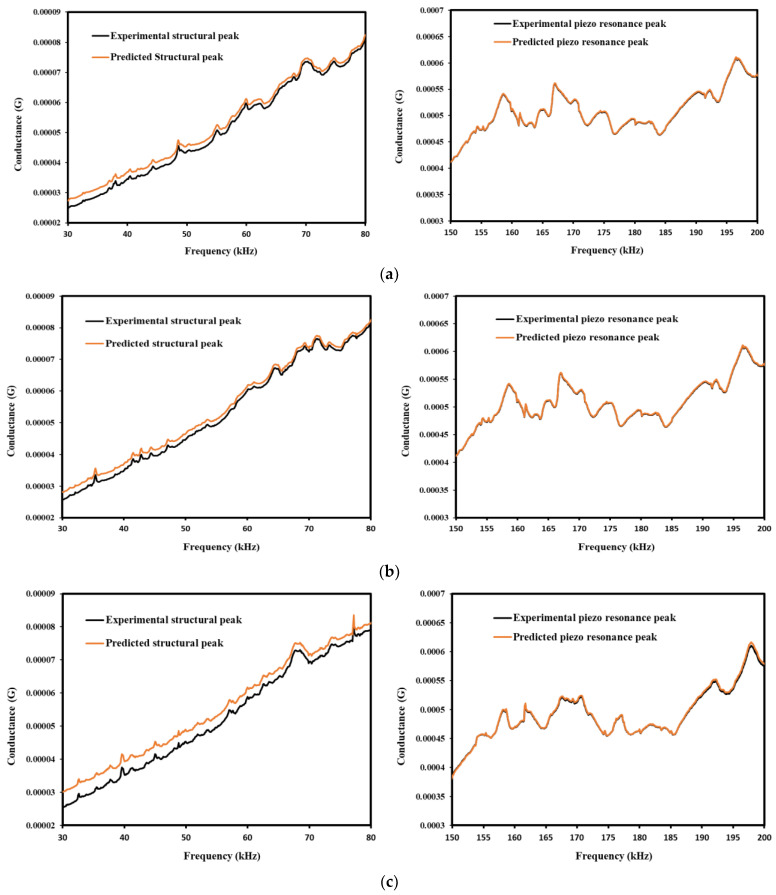

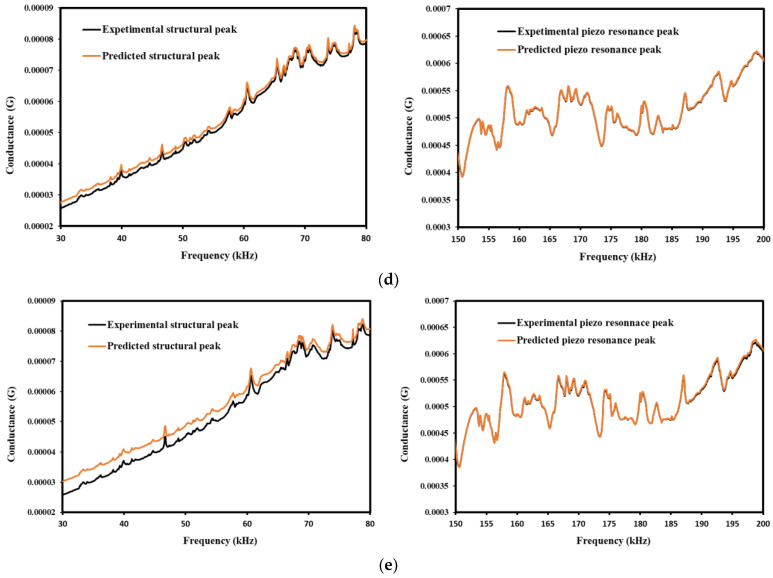
Comparison between experimental and predicted structural and Piezo resonance peak of (**a**) Baseline, (**b**) Strain Displacement @5 mm, (**c**) Strain Displacement @10 mm, (**d**) Strain Displacement @15 mm, (**e**) Strain Displacement @20 mm.

Overall, the above shown results indicates that the CNN-LSTM-based hybrid model performs better for the prediction of baseline signatures and piezo coupled signatures for different strain displacement range. In a practical scenario, where baseline data are not available, the deep learning-based hybrid model can be used to forecast the EMI signals for better simulation of preexisting data of structural information. Furthermore, this study is extended to forecast EMI signatures for the structural frequency range (30–80) kHz and piezo resonance peak (150–200) kHz for the baseline Figure 17a,b shows the proposed CNN-LSTM model to forecast the frequency range of the structural and piezo resonance frequency range for prediction of baseline signature. These structural peaks accurately depict the piezo coupled behavior of the specimen, a very essential element for the EMI technique. Similar studies have been reported by researchers in their work to investigate the hydration monitoring process using structural and piezo resonance peaks [103,104].

### 4.4. Statistical Validation of Predicted and Experimental Data

**Figure 18:**
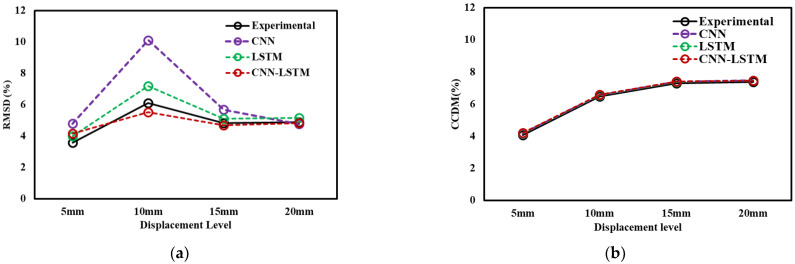
Comparison between experimental and predicted statistical indices (**a**) RMSD (**b**) CCDM.

Figure 18a compares the experimental and predicted deep learning-based RMSD values, simulated for the baseline signature. It is observed that the experimental and predicted RMSD follows increasing and similar trends with a small outliers at the 10 mm displacement level. However, the developed model follows similar patterns to those of experimental realizations. Figure 18b shows the variation of CCDM indices in experimental data and proposed deep learning models. The results of CCDM indices suggest that similar trends are being predicted as experimental CCDM indices, for higher strain displacement values raised for axial pull-out test with low error. In summary, the results show that CNN-LSTM hybrid model found to be effective for prediction of the healthy state of EMI signatures, called pristine or baseline signature and monitor structural health of reinforced concrete structures (e.g., strength, durability, and damage).

## 5. Conclusions 

This paper comprises the impedance-based structural diagnosis for a concrete and steel bond through a tensile pull-out test along with a sensor attached. The axial pull tests were carried to obtain static results, i.e., stress and slip. The bond strength between steel and concrete was evaluated and found satisfactory with elastic strain variation. Further, the piezo coupled signatures were obtained through a bonded PZT patch in a steel bar for a different strain hold, i.e., for a different displacement range recorded for LVDT attached in UTM. The statistical indices, RMSD and CCDM, reprising for the different stain ranges, have been plotted for easy interpretation. 

Further, the neural networks-based predictive models i.e., LSTM and CNN are utilized for better monitoring the bond strength and baseline condition of intended structure. The prediction baseline data are a much-needed parameter for statistical damage indication for EMI-based SHM. This study also includes the development of a novel hybrid model for accurate modeling of strength prediction for pristine condition and for various strain range. The developed model is implemented for the prediction of EMI signals subjected to pullout loading. The outcome of the proposed model is further verified with experimental results. The evaluation metrics (RMSE, MAE, and MAPE) for different prediction models are compared to highlight the sensitivity and accuracy of the proposed hybrid model (CNN-LSTM) towards bond strength prediction. From the results, it is found that the proposed CNN-LSTM algorithm represents a reliable and consistent tool for the prediction of baseline signatures.

Overall, the results of the present investigation shows a very encouraging and prominent application of EMI-based signature for interphase bond strength prediction for non-destructive evaluation (NDE) approach. The proposed CNN-LSTM algorithm represents a reliable and consistent tool for the prediction of baseline signatures. Future research should also consider the potential effects of hybrid models along with adequate hyper parameter optimization techniques to create even more accurate predictive models. The proposed model will be modified and optimized to enhance its performance in the prediction of complex structural damages. The deep learning algorithms are the most potent tool to extract the features for any time series changes of any physical system. Utilizing EMI technique helps the signature prediction and feature extraction, and their changes, for different parametric variations, accommodate the flexibility in decision making for long-term SHM. Having the model to analyze the impedance-based date will add more function to predict the baseline behaviour when most of the time primitive system data are missing. Furthermore, through inverse approach, this model can be utilized to understand the inherent structural changes of the specimen due to progressive damage.

## Figures and Tables

**Table 1:** Mix proportion of concrete.

W/C Ratio	Materials (kg/m^3^)			
**0.55**	**Cement**	**River Sand**	**Coarse Aggregates**	**Water**
	360	552	1303	198

**Table 2:** Material Properties of PZT patch.

Materials	Property	Unit	Value
PZT	Poisson’s ratio	µ	0.3000
	Piezoelectric strain coefficient	m/V	$-2.100\times 10^{-10}$
	Dielectric loss factor	δ	0.0224
	Mechanical loss factor	$Q_{m}$	0.0325
	Young’s modulus	$N/m^{2}$	$6.667\;\times \;10^{10}$
Steel bar	Density	Kg/m^3^	7850
	Yield stress	MPa	500
	Elongation	%	18
	Ultimate tensile stress	MPa	580
Adhesive	Flexural strength	MPa	61
	Flexural modulus	MPa	4354.9
	Tensile strength	MPa	26

**Table 3:** Hyper-parameters used for Deep Learning Models.

LSTM Model		CNN Model	
Hyper-parameters	Value	Hyper-parameters	Value
Learning Rate	0.031	Learning Rate	0.054
Dropout Rate	0.065	Dropout Rate	0.032
No of Hidden Layers	1	No of Hidden Layers	2
Neurons Per Layer	175	Kernel size	3
Batch Size	64	Batch Size	128
Activation Function	tanh	Activation Function	relu

**Table 4:** Performance Metrics.

Metrics	Formula
RMSE	$\sqrt{\frac{\sum_{i=1}^{n}{\left(y_{i}-{\hat{y}}_{i}\right)}^{2}}{n}}$
MAE	$\frac{\sum_{i=1}^{n}\left|y_{i}-{\hat{y}}_{i}\right|}{n}$
MAPE	$\frac{100}{n}\overset{n}{\underset{i=1}{\sum }}\left|\frac{y_{i}-{\hat{y}}_{i}}{y_{i}}\right|$
$y_{i}$: Actual or observed value ${\hat{y}}_{i}$: Predicted value *n*: No of data points	

**Table 5:** Accuracy of proposed CNN-LSTM model for Sample 1,2.

Model and Metric	Sample 1			Sample 2		
	MAE	RMSE	MAPE	MAE	RMSE	MAPE
Baseline	0.0000090	0.0000141	0.87	0.0000219	0.0000351	1.89
Strain Displacement @5 mm	0.0000257	0.0000277	2.32	0.0000304	0.0000612	2.03
Strain Displacement @10 mm	0.0000162	0.0000175	1.53	0.0000176	0.0000225	1.84
Strain Displacement @15 mm	0.0000088	0.0000115	0.79	0.0000182	0.0000293	1.59
Strain Displacement @20 mm	0.0000081	0.0000117	0.74	0.0000291	0.0000347	2.12

**Table 6:** Accuracy of deep learning models for Sample 1.

Model and Metric	LSTM			CNN		
	MAE	RMSE	MAPE	MAE	RMSE	MAPE
Baseline	0.0000758	0.0000789	6.82	0.0000785	0.0000821	7.01
Strain Displacement @5 mm	0.0000591	0.0000611	5.31	0.0000456	0.0000483	4.05
Strain Displacement @10 mm	0.0000588	0.0000608	5.49	0.0000611	0.0000644	5.64
Strain Displacement @ 15 mm	0.0000650	0.0000677	5.79	0.0000487	0.0000514	4.31
Strain Displacement @ 20 mm	0.0000691	0.0000717	6.19	0.0000637	0.0000670	5.67

**Table 7:** Accuracy deep learning models for Sample 2.

Model and Metric	LSTM			CNN		
	MAE	RMSE	MAPE	MAE	RMSE	MAPE
Baseline	0.0000361	0.0000490	3.18	0.0000402	0.0000672	2.98
Strain Displacement @5 mm	0.0000365	0.0000455	3.42	0.0000276	0.0000468	3.42
Strain Displacement @10 mm	0.0000354	0.0000469	3.14	0.0000330	0.0000651	2.22
Strain Displacement @15 mm	0.0000226	0.0000283	2.35	0.0000375	0.0000667	2.64
Strain Displacement @20 mm	0.0000251	0.0000322	2.51	0.0000317	0.0000526	2.40

## Data Availability

Not applicable.
